# pH Effect and Chemical Mechanisms of Antioxidant Higenamine

**DOI:** 10.3390/molecules23092176

**Published:** 2018-08-29

**Authors:** Yulu Xie, Xican Li, Jingyu Chen, Yuman Deng, Wenbiao Lu, Dongfeng Chen

**Affiliations:** 1 School of Chinese Herbal Medicine, Guangzhou University of Chinese Medicine, Waihuan East Road No. 232, Guangzhou Higher Education Mega Center, Guangzhou 510006, China; xieyulu1900@163.com (Y.X.); Chenjingyu1102@163.com (J.C.); qyfan@gzucm.edu.cn (Y.D.); luwb1@gzucm.edu.cn (W.L.); 2 Innovative Research & Development Laboratory of TCM, Guangzhou University of Chinese Medicine, Waihuan East Road No. 232, Guangzhou Higher Education Mega Center, Guangzhou 510006, China; 3 School of Basic Medical Science, Guangzhou University of Chinese Medicine, Waihuan East Road No. 232, Guangzhou Higher Education Mega Center, Guangzhou 510006, China; 4 Research Center of Basic Integrative Medicine, Guangzhou University of Chinese Medicine, Waihuan East Road No. 232, Guangzhou Higher Education Mega Center, Guangzhou 510006, China

**Keywords:** higenamine, antioxidant, mechanistic chemistry, pH effect

## Abstract

In this article, we determine the pH effect and chemical mechanism of antioxidant higenamine by using four spectrophotometric assays: (1) 2-phenyl-4,4,5,5-tetramethylimidazoline-1-oxyl-3-oxide radical (PTIO•)-scavenging assay (at pH 4.5, 6.0, and 7.4); (2) Fe^3+^-reducing power assay; (3) Cu^2+^-reducing power assay; and (4) 1,1-diphenyl-2-picryl-hydrazyl (DPPH•)-scavenging assay. The DPPH•-scavenging reaction product is further analyzed by ultra-performance liquid chromatography, coupled with electrospray ionization quadrupole time-of-flight tandem mass spectrometry (UPLC-ESI-Q-TOF-MS/MS) technology. In the four spectrophotometric assays, higenamine showed good dose-response curves; however, its IC_50_ values were always lower than those of Trolox. In UPLC-ESI-Q-TOF-MS/MS analysis, the higenamine reaction product with DPPH• displayed three chromatographic peaks (retention time = 0.969, 1.078, and 1.319 min). The first gave *m*/*z* 541.2324 and 542.2372 MS peaks; while the last two generated two similar MS peaks (*m*/*z* 663.1580 and 664.1885), and two MS/MS peaks (*m*/*z* 195.9997 and 225.9971). In the PTIO•-scavenging assays, higenamine greatly decreased its IC_50_ values with increasing pH. In conclusion, higenamine is a powerful antioxidant—it yields at least two types of final products (i.e., higenamine-radical adduct and higenamine-higenamine dimer). In aqueous media, higenamine may exert its antioxidant action via electron-transfer and proton-transfer pathways. However, its antioxidant action is markedly affected by pH. This is possibly because lower pH value weakens its proton-transfer pathway via ionization suppression by solution H^+^, and its electron-transfer pathway by withdrawing the inductive effect (-I) from protonated N-atom. These findings will aid the correct use of alkaloid antioxidants.

## 1. Introduction

Natural phenolic antioxidants comprise a wide range of oxygen-containing organic compounds such as phenolic acids, flavonoids, chalcones, and stilbenes. Phenolic-OH is an indispensable moiety in these molecules. In addition, there are other O-containing moieties such as –OR (alkoxyl group), –O– (ether bond), –COOH (carboxyl group), –COOR (ester group), –C=O (carbonyl group), and even sugar residue [1,2,3,4,5]. Undoubtedly, the existence of O-atom makes these phenolic antioxidants acidic or neutral. 

Alkaline phenolic antioxidants have seldom been reported [6]. Typical alkaloids are nitrogen-containing heterocyclic compounds from plants (especially Chinese herbal medicines). According to the heterocycle skeleton, these alkaloids can be classified into 26 types such as isoquinoline-type, acridone-type, lycopodine-type, pyrrolidine-type, and quinolizidine-type. Among the first three types, phenolic-OH is documented to occasionally attach to alkaline skeletons to construct phenolic alkaloids [5]. 

Phenolic alkaloids and other phenolics have been reported to be mediated by proton-transfer (PT, i.e., H^+^-transfer) to display antioxidant action [6,7,8,9]. Proton-transfer is highly associated with H^+^ ionization from phenolic-OH, especially in protic solvents [10]. For alkaloids, H^+^ is a highly sensitive species; the nitrogen atom (N) in phenolic alkaloid combines with H^+^ to form an amine salt. Therefore, phenolic alkaloid is protonated at the N-atom. The protonated N-atom bears positive charge and thus has strong withdrawing electron ability. The change in electron density affects antioxidant levels because electron-transfer (ET) ability is an important factor of an antioxidant [9]. This predicts that there are possibly multiple pH effects towards antioxidant alkaloids. Until now, no study has focused on pH effect towards antioxidant alkaloids, despite the fact that there have been reports on acidic antioxidants (such as ferulic acid, protocatechuic acid [1], tannic acid [11], and caffeic acid [12]), neutral antioxidants (e.g., peptides [13]), and amphoteric antioxidants (e.g., folates [14]). 

**Figure 1:**
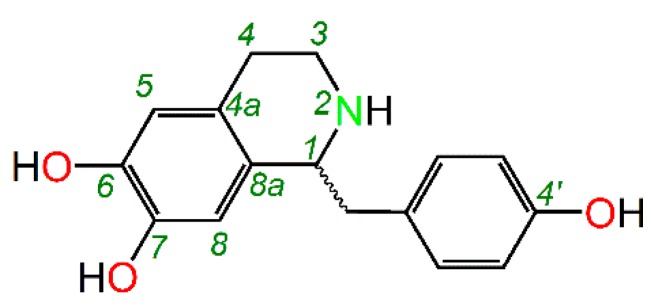
The structure of higenamine (﹏, uncertain stereo configuration).

This study tries to use higenamine as representative of phenolic alkaloids. As shown in Figure 1, higenamine is an isoquinoline-type alkaloid bearing three phenolic-OHs (i.e., 6,7,4′-tri-OHs). Hence, higenamine is a representative phenolic alkaloid. As an alkaloid, higenamine is reported to have various pharmacological properties, such as anti-thrombotic, anti-apoptotic, anti-inflammatory, and immunomodulatory effects [15]. According to free radical biology and medicine, these pharmacological properties are associated with antioxidative effects [16,17]. For instance, higenamine decreases the release of cytochrome c (a cellular oxidative promotor), and decreases apoptotic cell death in myocardial ischemia-reperfused rats [18]. These clues indicate that higenamine has antioxidant activity and is an ideal representative for pH effect study. 

The possible pH effect undoubtedly depends on antioxidant mechanism. Therefore, the mechanisms of antioxidant higenamine were studied using spectrophotometric method and ultra-performance liquid chromatography coupled with electrospray ionization quadrupole time-of-flight tandem mass spectrometry (UPLC-ESI-Q-TOF-MS/MS) method. These studies will help understand the pH effect in phenolic alkaloids and the characteristics of alkaloid antioxidants. Particularly, these findings concerning pH effect caution us on the clinical application of antioxidant alkaloids (e.g., vincristine-loaded liposome [19]). 

## 2. Results and Discussion

The initial estimation of the antioxidant potential of higenamine was performed by 1,1-diphenyl-2-picryl-hydrazyl (DPPH•, Supplementary 1) scavenging assay a—popular biochemistry method. As seen in Supplementary 2, higenamine concentration dependently increased the DPPH•-scavenging percentage. However, its IC_50_ value was lower than that of Trolox (Table 1). These results suggested higenamine was a powerful antioxidant. 

Like other radical reactions, DPPH•-scavenging by higenamine is also a chain-reaction, which at least includes a propagation step and termination step [20]. The termination step actually involves decay of the radical (or its intermediates) [21]. During this step, the radicals (or its intermediates) are covalently linked to each other form a stable adduct product, and thus to terminate the reaction [22]. Hence, these final products are usually radical adducts, mainly including antioxidant-radical adduct and antioxidant-antioxidant dimer. Accordingly, the reaction is termed as radical adduct formation (RAF) reaction [21,23]. Some literature also called the RAF products as non-radical products [10,24]. Strictly, non-radical products also comprise the Diels-Alder product, a newly found final product not based on the radical adduct [25]. To test RAF possibility, the product mixture of higenamine and DPPH• was determined using UPLC-ESI-Q-TOF-MS/MS technology in this study. 

**Figure 2:**
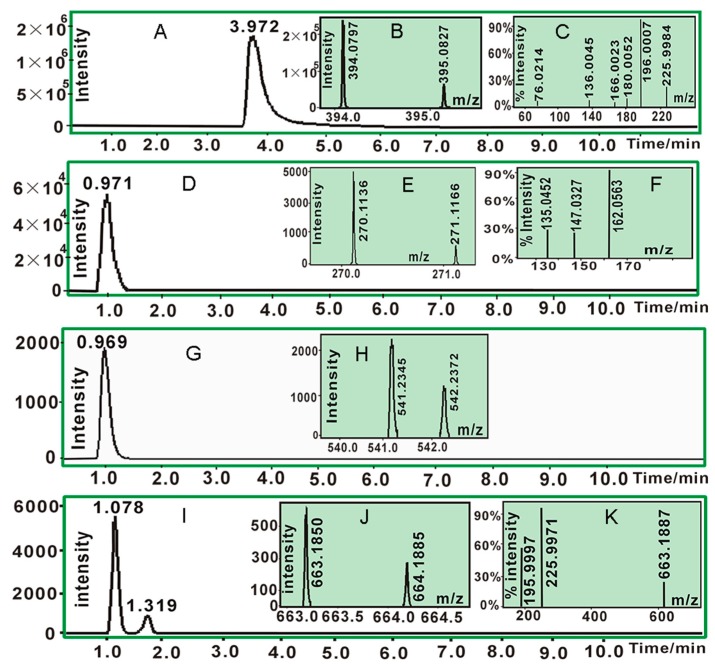
The main results of UPLC-ESI-Q-TOF-MS/MS analysis: (**A**). Chromatogram of 1,1-diphenyl-2-picryl-hydrazl radical *(*DPPH•) when the formula [C_18_H_12_N_5_O_6_] was extracted; (**B**). MS spectrum C of DPPH•; (**C**). MS/MS spectrum of DPPH•; (**D**). chromatogram of higenamine when the formula [C_16_H_17_NO_3_] was extracted; (**E**). MS spectrum of higenamine; (**F**). MS/MS spectrum of higenamine; (**G**). chromatogram of higenamine-higenamine when the formula [C_32_H_32_N_2_O_6_-H]^−^ was extracted; (**H**). MS spectrum of higenamine-higenamine (MS/MS spectrum of higenamine-higenamine could not be found); (**I**). chromatogram of non-radical product of higenamine-DPPH when the formula [C_34_H_28_N_6_O_9_-H]^−^ was extracted; (**J**). MS spectrum of the non-radical product of higenamine-DPPH; (**K**). MS/MS spectrum of higenamine-DPPH). (The original data are detailed in Supplementary 3).

**Figure 3:**
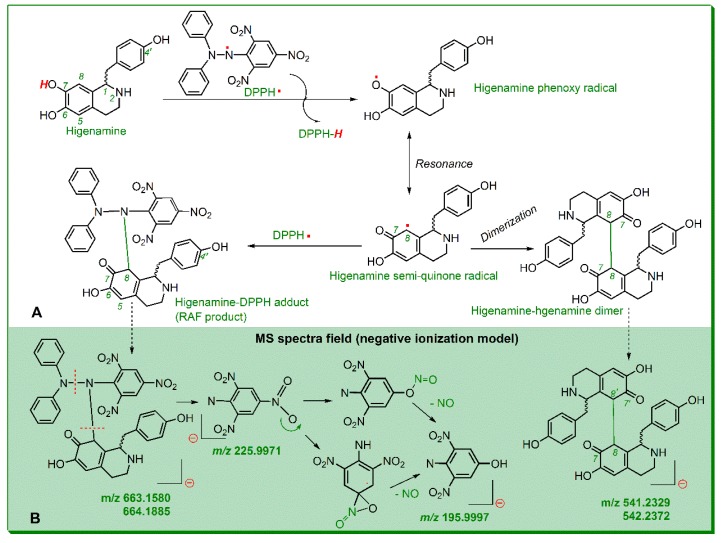
Possible radical-adduct-formation (RAF) reactions between higenamine and 1,1-diphenyl-2-picryl-hydrazl radical (DPPH•) (**A**), and MS spectral elucidations (**B**).

As suggested by UPLC-ESI-Q-TOF-MS/MS analysis, the DPPH• standard (RT = 3.972 min, Figure 2A) yielded molecular ion peaks (*m*/*z* 394.0797 and 395.0827) in MS spectrum (Figure 2B) and two fragments (*m*/*z* 196.0007 and 225.9984) in MS/MS spectrum (Figure 2C). Higenamine standard (RT = 0.971 min, Figure 2D) gave rise to molecular ion peaks (*m*/*z* 270.1136 and 271.1166) in MS spectrum (Figure 2E) and three fragments (*m*/*z* 135.0452, 147.0327, and 162.0563) in MS/MS spectrum (Figure 2F). Their product, however, gave three chromatographic peaks (RT = 0.969, 1.078, and 1.319 min, Figure 2G,K). In the MS spectral field, the first chromatographic peak was broken into two MS peaks (*m*/*z* 541.2324 and 542.2372, Figure 2H). The *m*/*z* values were twice the molecular weight of higenamine, suggesting generation of dimeric higenamine-higenamine. The latter two chromatographic peaks similarly generated two MS peaks (*m*/*z* 663.1580 and 664.1885, Figure 2J). The values were equivalent to the total molecular weight of DPPH• *plus* higenamine. Therefore, it could be proposed that RAF reaction took place between DPPH• and higenamine. The proposal was further confirmed by the fragments *m*/*z* 195.9997 and 225.9971 from the MS/MS spectrum (Figure 2K), which were also found in the DPPH• standard (Figure 2C). Based on MS spectral data and previous work [25,26,27], RAF reactions between DPPH• and higenamine can be proposed as in Figure 3A; while the MS spectral elucidation is described in Figure 3B. 

In short, as a powerful antioxidant, higenamine effectively scavenged DPPH• and gave rise to two types of final and stable products, i.e., higenamine-DPPH adduct and higenamine-higenamine dimer. It is worth mentioning that in higenamine-DPPH adduct and higenamine-higenamine dimer (Figure 3A), the covalent bonds were also linked to other positions, such as the 5-position of higenamine. Therefore, there were multiple chromatographic peaks in their adduct products (Figure 2I). 

From the aspect of biological relevance, DPPH•-scavenging assay is actually not an ideal model because the DPPH•-scavenging assay is performed in alcoholic solution (not aqueous solution). Furthermore, DPPH•-scavenging reaction is involved in a series of antioxidant mechanisms including hydrogen atom transfer (HAT) [28], sequential electron proton transfer (SEPT) [8], proton-coupled electron transfer (PCET) [29], and proton loss single electron transfer (SPLET) [30]. All these antioxidant mechanisms could essentially be classified as two main reactions, i.e., ET and PT [31]. The DPPH•-scavenging model could not give mere ET or PT, and thus, it could hardly lead to a clear conclusion of antioxidant mechanism. 

Recently, our team developed a new antioxidant assay, the 2-phenyl-4,4,5,5-tetramethylimidazoline-1-oxyl 3-oxide radical (PTIO•)-scavenging assay. PTIO• is an oxygen-centered radical with two oxidized N-atoms (Supplementary 1), and can be protonated at the O-atom (not N-atom) [32]. The situation is similar to cellular reactive oxygen species (ROS). Cellular ROS can be protonated at the O-atom. For instance, •O_2_^−^ can be protonated to HO_2_•. Besides, the assay is conducted in aqueous solution, and hence the PTIO•-scavenging assay has more biological relevance (especially at pH 7.4). 

As shown in Supplementary 4, higenamine dose-dependently increased the PTIO•-scavenging percentage at pH 7.4. Its IC_50_ value was lower than that of Trolox (Table S2). These indicated that higenamine was a powerful antioxidant that could effectively scavenge oxygen-centered radicals in aqueous media. This is similar to cellular ROS-scavenging reactions (e.g., •OH-scavenging, •O_2_^−^-scavenging, and ROO•-scavenging) [13,33]. 

Below pH 5.0, PTIO•-scavenging action was proven to be a mere ET reaction by cyclic voltammetry [32]. As shown in Supplementary 4, higenamine could scavenge PTIO• in a dose-dependent manner at pH 4.5, suggesting a mere ET potential of antioxidant higenamine. The ET potential was further supported by evidence from Fe^3+^-reducing power assay (pH 3.6) and Cu^2+^-reducing assay (pH 7.4). As seen in Supplementary 5 and Table S3, higenamine increased FRAP and Cu^2+^-reducing power percentages with increase in concentration. The two metal-reducing power assays were suggested to be ET reactions [21,34,35,36]. Therefore, it could be inferred that ET played a role during the antioxidant action of higenamine. 

**Figure 4:**
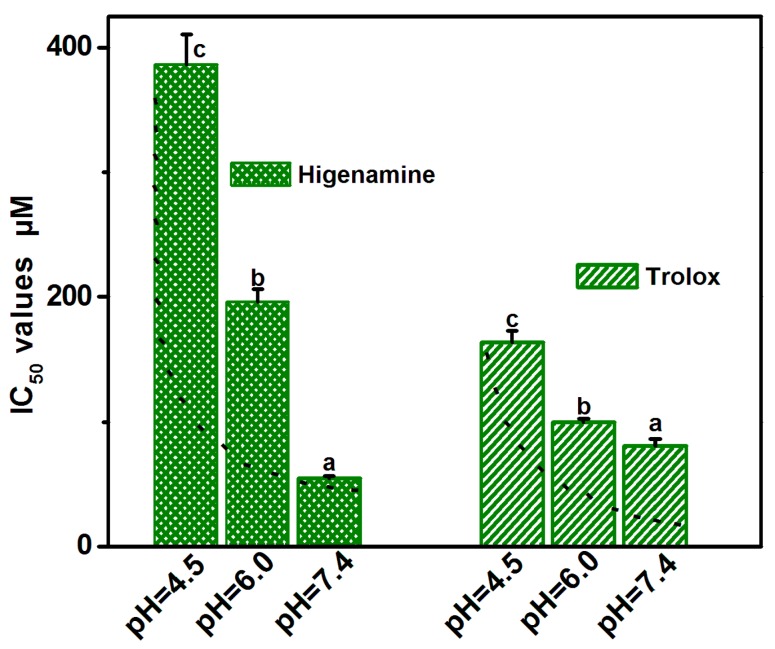
IC_50_ values of higenamine and Trolox in 2-phenyl-4,4,5,5-tetramethylimidazoline-1-oxyl 3-oxide radical (PTIO•)-scavenging assay at pH 4.5, 6.0 and 7.4. Means values with different superscripts (a or b or c) for the same sample are significantly different (*p* < 0.05).

To test PT potential, higenamine was further determined by PTIO•-scavenging capacity at pH 6.0 (Supplementary 4). The IC_50_ value at pH 6.0 was then compared to those at pH 7.4 and pH 4.5. The comparison revealed that from pH 7.4 to pH 6.0 and from pH 7.4 to pH 4.5, higenamine increased its IC_50_ values 3.57 times (196.6/55.0) and 7.02 times (386.5/55.0), respectively (Figure 4 and Table S2). It was then clear that there was a strong pH effect. Particularly, lower pH values severely suppressed higenamine’s antioxidant action. Furthermore, a strong pH effect meant that the PTIO•-scavenging action of higenamine was mediated by PT (i.e., H^+^-transfer). It agreed with the previous findings, that PT could also be found in the antioxidant process of alkaloid sinapine [8], and even in enzymatic biochemical reactions [37]. 

Higenamine had ET and PT potentials during its antioxidant action in aqueous media. In fact, both ET and PT played important roles in the interaction of ROO• with phenolic antioxidants in aqueous media [13].

It should be emphasized that the strong pH effect itself was an important characteristic of antioxidant higenamine. The strong pH effect could also be found in two N-containing antioxidants kukoamines A and B [6]. However, the pH effect of O-containing antioxidants was generally mild. A typical example was Trolox, a standard antioxidant with -COOH. As seen in Figure 4, from pH 4.5 to pH 7.4 and from pH 6.0 to pH 7.4, Trolox antioxidant levels increased by only 2.0 times and 1.2 times, respectively. Similar mild pH effect was observed in the 3,5-di-*O*-caffeoylquinic acid family, which even slightly decreased the antioxidant level 0.93 times (108.6/116.5) from pH 4.5 to pH 7.4 [38]. The difference suggests that N-containing antioxidant is more sensitive to lower pH values than O-containing antioxidant; and thus, the strong pH effect might be closely associated with the existence of N-atom. 

**Figure 5:**
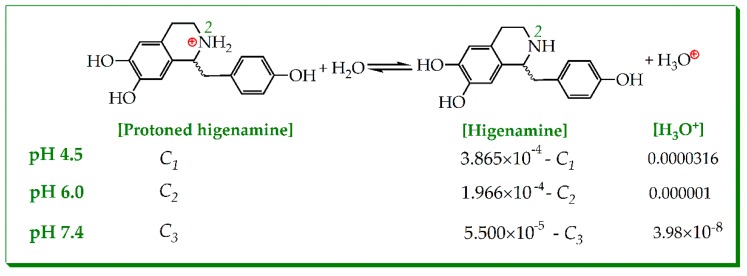
Ionization equilibrium of protonated higenamine (the total concentration of higenamine was adopted from its IC_50_ values listed in Table S2).

In higenamine molecule, the alkaline N-atom (i.e., 2-N) was at the tetrahydroisoquinolin fused-ring (Figure 1). Acidic buffer (lower pH value) exposed the solution H^+^ to 2-N to form protonated higenamine (i.e., its conjugate acid). Reversibly, the protonated higenamine gave H^+^ through the ionization equilibrium (Figure 5). However, there was no available acidic ionization equilibrium (Ka value) of higenamine. Roughly, its alkalinity was equivalent to that of 1,2,3,4-tetrahydroisoquinoline. However, the 1,2,3,4-tetrahydroisoquinoline derivatives varied in pKa values from 8–9 (at 25 °C) [39]. Considering the steric hinderance of 1-C, the pKa value of protonated higenamine was lower. Given pKa = 8.0 (Ka = 10^−8^) and total concentrations of higenamine were IC_50_ values (3.865 × 10^−4^ M, 1.966 × 10^−4^ M, and 5.50 × 10^−5^ M, respectively, at pH 4.5, 6.0, and 7.4, Figure 4 and Table S2), the ionization equilibrium of higenamine could be described as in Figure 5.

(1) $$\mathrm{Ka}=\frac{\left[Higenamine\right]\times \left[H_{3}O^{+}\right]}{\left[Protoned\;higenamine\right]}=1.0\times 10^{8}$$

**Figure 6:**
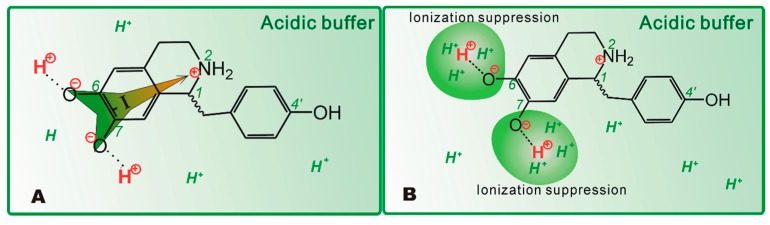
The two approaches for the pH effect towards antioxidant higenamine: (**A**). withdrawing inductive effect (-I) reduced the electron-transfer (ET) potential of benzene-ring; (**B**). suppressed ionization of phenolic-OH by solution H^+^.

In terms of the equilibrium constant equation (Equation (1)), the concentrations of protonated higenamine were calculated as: C_1_ ≈ 3.859 × 10^−4^ M; C_2_ ≈ 1.947 × 10^−4^ M; C_3_ ≈ 4.39 × 10^−5^ M. This meant that the protonated levels of higenamine were approximately 99.97%, 99.01%, and 79.81% in buffers of pH 4.5, 6.0, and 7.4, respectively. Apparently, the protonation level of higenamine deepened with decrease in pH value. At lower pH value, a high-level of protonated higenamines meant high-level positive charge in 2-N (Figure 6). The positive charge in 2-N strongly withdrew electrons from the benzene-ring (especially the left one, Figure 6A). The strong withdrawing effect (-I) reduced the electron-density of the benzene-ring to weaken the ET-potential of phenolic-OH.

On the other hand, the massive solution H^+^ (i.e., lower pH value) suppressed ionization (i.e., PT from phenolic-OH, Figure 6B), to lower its PT potential. In aqueous buffer with low pH value, solution H^+^ weakened the PT potential via suppressing ionization; while the protonated N-atom weakened the ET potential via the -I effect. Such synergistic effect greatly decreased the antioxidant activity of higenamine. However, it is worth mentioning that: (1) The pKa value herein was not precise, and thus, it was hard to judge which approach predominated, the -I effect or ionization suppression. Nevertheless, the conclusion that both -I and ionization suppression synergistically affect the action of the alkaloid antioxidant is basically accepted. (2) Our findings may also be used to partly explain the pH effect in the 2,2′-azinobis(3-ethylbenzthiazoline-6-sulfonic acid) ion radical (ABTS•^+^)-scavenging assay [1,13,40]. As seen in Supplementary 1, the ABTS•^+^ radical bears two tertiary amine moieties and two Schiff base moieties, which can accept H^+^ to yield protonated N-atoms. And thus ABTS•^+^ radical can be regarded as a special alkaloid. The pH effect may be from the protonation of N-atom in ABTS•^+^. 

Finally, quantitative analysis based on IC_50_ ratio values revealed that compared to Trolox, higenamine possessed 1.4×, 1.5×, 2.1×, and 2.7× relative antioxidant levels in the DPPH•-scavenging assay, PTIO•-scavenging assay (pH 7.4), Fe^3+^-reducing assay, and Cu^2+^-reducing assay, respectively (Table 1). On average, higenamine possessed 1.9× antioxidant level relative to Trolox. It further suggested that higenamine was a powerful antioxidant. 

## 3. Materials and Methods 

### 3.1. Chemicals 

Higenamine (CAS 5843-65-2, C_16_H_17_NO_3_, M.W. 271.3, ≥97%, Supplementary 6) was obtained from Chengdu Biopurify Phytochemicals Ltd. (Chengdu, China). 1,1-Diphenyl-2-picryl-hydrazl radical (DPPH•), (±)-6-hydroxyl-2,5,7,8-tetramethlychromane-2-carboxylic acid (Trolox), and 2,9-dimethyl-1,10-phenanthroline (neocuproine) were purchased from Sigma-Aldrich Shanghai Trading Co. (Shanghai, China). 2-Phenyl-4,4,5,5-tetramethylimidazoline-1-oxyl-3-oxide radical (PTIO•) and 2,4,6-tripyridyltriazine (TPTZ) were from TCI Chemical Co. (Shanghai, China). All other reagents were of analytical grade. 

### 3.2. DPPH•-Scavenging Assay (Methanol Solution)

DPPH•-scavenging activity was determined as previously described [17]. Briefly, 80 μL DPPH• solution (0.1 mol/L) was mixed with the indicated concentration of the sample (0.083 mg/mL, 2–10 μL) dissolved in methanol. The mixture was maintained at room temperature for 30 min, and absorbance was measured at 519 nm on a spectrophotometer (Unico 2100, Shanghai, China). The percentage of DPPH•-scavenging activity was calculated as follows:

$$\mathrm{Inhibition}\;\%=\frac{A_{0}-A}{A_{0}}\;\times 100\%$$

where A_0_ is the absorbance of the control without the sample and A is the absorbance of the reaction mixture with the sample.

### 3.3. UPLC-ESI-Q-TOF-MS/MS Analysis of DPPH• Reaction Products with Higenamine

The reaction conditions were based on kukoamine A [6]. Briefly, methanol solution of higenamine was mixed with a solution of DPPH• radical in methanol in molar ratio 1:2, and the resultant mixture was incubated for 10 h at room temperature. The product was then filtered through a 0.22 μm filter for UPLC-ESI-Q-TOF-MS/MS analysis

UPLC-ESI-Q-TOF-MS/MS analysis was based on the method described in our previous study [23]. The UPLC-ESI-Q-TOF-MS/MS analysis system was equipped with a C_18_ column (2.0 mm i.d. × 100 mm, 2.2 μm, Shimadzu Co., Kyoto, Japan). The mobile phase used for elution of the system consisted of a mixture of acetonitrile (phase A) and 0.1% formic acid / water (phase B). The column was eluted at flow rate 0.2 mL/min with the following gradient elution program: 0–2 min, maintain 30% B; 2–10 min, 0–30% B; 10–12 min, 0–30% B. The sample injection volume was set at 1 μL to separate different components. Q-TOF-MS/MS analysis was performed on a Triple TOF 5600*^plus^* Mass spectrometer (AB SCIEX, Framingham, MA, USA) equipped with ESI source run in the negative ionization mode. The scan range was set at 100–2000 Da. The system was run with the following parameters: ion spray voltage, −4500 V; ion source heater, 550 °C; curtain gas (CUR, N_2_), 30 psi; nebulizing gas (GS1, air), 50 psi; Tis gas (GS2, air), 50 psi. The declustering potential (DP) was set at −100 V, whereas the collision energy (CE) was set at −40 V with collision energy spread (CES) 20 V. The non-radical products were quantified by extracting corresponding formulae (e.g., [C_34_H_28_N_6_O_9_-H]^-^ for higenamine-DPPH•) from the total ion chromatogram and integrating the corresponding peak.

### 3.4. pH Effect of Higenamine on PTIO•-Scavenging Ability 

The PTIO•-scavenging assay was conducted based on our method [33]. Briefly, higenamine sample solution (*x* = 0–20 μL, 1 mg/mL) was added to (20−*x*) μL 95% ethanol, followed by 80 μL aqueous PTIO• solution. The aqueous PTIO• solution was prepared using phosphate-butter solution (0.1 mM, pH 4.5). The mixture was maintained at 37 °C for 2 h; absorbance was then measured at 560 nm using the aforementioned microplate reader. The PTIO• inhibition percentage was presented in Section 3.2.

The above experimental protocol was repeated using pH 6.0 buffer and pH 7.4 buffer. 

### 3.5. Ferric-Reducing Antioxidant Power (pH 3.6)

The assay was adapted from Benzie and Strain [35]. Briefly, the test sample (*x* = 2–10 μL, 0.15 mg/mL) was added to (10−*x*) μL of 95% ethanol to prepare sample solution. Then, 10 mM TPTZ, 20 mM FeCl_3_, and 0.25 M acetate buffer were mixed at 1:1:10 (pH 3.6) to prepare detection reagent. The freshly prepared detection reagent (90 μL) was taken out and added to the sample solution. The total volume of the reaction system was 100 μL. After 30 min incubation at ambient temperature, the reaction mixture was measured at 593 nm using distilled water as blank. The reducing power of the sample relative to the maximum absorbance was calculated using the following formula:

$$\mathrm{Relative}\;\mathrm{reducing}\;\mathrm{effect}\%=\frac{A-A_{\min}}{A_{\max}-A_{\min}}\times 100\%$$

where A_min_ is the absorbance of the control without sample, A is the absorbance of the reaction mixture with sample, and A_max_ is the maximum absorbance of the reaction mixture with sample. 

### 3.6. Cu^2+^-Reducing Assay (pH 7.4)

The reducing power capacities of cupric ions (Cu^2+^) were measured according to a previously described method with slight modification [9]. Briefly, 12 μL CuSO_4_ aqueous solution (10 mmol/L), 12 μL neocuproine ethanolic solution (7.5 mmol/L), and (76−*x*) μL CH_3_COONH_4_ buffer solution (0.1 mol/L, pH 7.4) were added to test tubes with different volumes of sample (0.15 mg/mL, *x* = 4–20 μL). The total volume was adjusted to 100 μL with buffer and mixed vigorously. The absorbance against a buffer blank was measured at 450 nm after 30 min. Increase in absorbance of the reaction mixture indicated increase in reduction capability. The relative reducing power was calculated using the formula described in Section 3.5. 

### 3.7. Statistical Analysis

Each experiment was performed in triplicate and the data reported as mean ± SD (standard deviation). Dose response curves were plotted using Origin 6.0 software (OriginLab, Northampton, MA, USA). The IC_50_ value was defined as the final concentration for 50% radical inhibition (relative reducing power or binding effect). Statistical comparisons were made by one-way analysis of variance (ANOVA) to detect significant differences using SPSS 13.0 (SPSS Inc., Chicago, IL, USA) for Windows. *p* < 0.05 was considered to indicate statistical significance. 

## 4. Conclusions 

Higenamine is a powerful antioxidant. Through antioxidant reaction, higenamine gave two types of final products, higenamine-radical adduct and higenamine-higenamine dimer. In aqueous media, its antioxidant action was fulfilled, probably via PT and ET pathways. Both pathways were greatly affected by pH value. Lower pH value could lower the PT potential via ionization suppression. Lower pH value could also weaken the ET potential via the -I effect of protonated N-atoms.

## Figures and Tables

**Table 1:** IC_50_ values of higenamine and Trolox in various antioxidant assays.

Assay	Higenamine μM	Trolox μM	IC_50,Trolox_/IC_50,higenamine_	
DPPH•-scavenging assay	17.1 ± 0.2 ^a^	23.3 ± 0.3 ^b^	1.4	Average 1.9
PTIO•-scavenging assay (pH 7.4)	55.0 ± 1.7 ^a^	81.0 ± 5.6 ^b^	1.5	
Fe^3+^-reducing power assay	30.0 ± 0.3 ^a^	63.1 ± 8.0 ^b^	2.1	
Cu^2+^-reducing power assay	57.0 ± 1.0 ^a^	154.8 ± 2.0 ^b^	2.7	

IC_50_ value was defined as the concentration of 50% effect percentage and expressed as mean ± SD (*n* = 3). Means values with different superscripts (a or b) in the same row were significantly different (*p* < 0.05). Trolox acted as positive control. The dose-response curves and IC_50_ calculation are listed in Supplementary 2, 4–5. Abbreviations: DPPH•, 1,1-diphenyl-2-picryl-hydrazl radical; PTIO•, 2-phenyl-4,4,5,5-tetramethylimidazoline-1-oxyl 3-oxide radical.

## Supplementary Materials

The following are available online. Supplementary 1: The structures of ABTS•+, PTIO•, and DPPH·; Supplementary 2: The experimental results of DPPH•-scavenging assay; Supplementary 3: The original data of UPLC-ESI-Q-TOF-MS/MS analysis; Supplementary 4: The experimental results of PTIO• assay; Supplementary 5: The experimental results of Fe^3+^-reducing and Cu^2+^-reducing assays. Supplementary 6: The analysis certificate of higenamine.

## Abbreviations

The following abbreviations are used in this manuscript:

ABTS	2,2′-azinobis(3-ethylbenzthiazoline-6-sulfonic acid)
DPPH•	1,1-diphenyl-2-picryl-hydrazl radical
ET	electron-transfer
HAT	hydrogen atom transfer
PT	proton-transfer
PTIO•	2-phenyl-4,4,5,5-tetramethylimidazoline-1-oxyl 3-oxide radical
ROS	reactive oxygen species
RNS	reactive nitrogen species
RAF	radical-adduct-formation
SD	standard deviation
TPTZ	2,4,6-tris(2-pyridyl-s-triazine)
Trolox	(±)-6-hydroxyl-2,5,7,8-tetramethlychromane-2-carboxylic acid
