# Supplementary material for: pH Effect and Chemical Mechanisms of Antioxidant Higenamine
Source: Molecules. 2018 Aug 29;23(9):2176. doi: 10.3390/molecules23092176 (PMC6225313; doi:10.3390/molecules23092176)
Supplement: Supplementary file 1 [file molecules-23-02176-s001.zip › molecules-347182-SI.pdf]

Supplements

*p*H Effect and Chemical Mechanisms of Antioxidant Higenamine

Yulu Xie[1,2], Xican Li [1, 2, *], Jingyu Chen[1,2], Yumang Deng[1,2], Dongfeng Chen [3, 4, *]

[1] School of Chinese Herbal Medicine, Guangzhou University of Chinese Medicine, Waihuan East Road No. 232, Guangzhou Higher Education Mega Center, Guangzhou 510006, China; xieyulu1900@163.com (Y.C.);

[2] Innovative Research & Development Laboratory of TCM, Guangzhou University of Chinese Medicine, Waihuan East Road No. 232, Guangzhou Higher Education Mega Center, Guangzhou 510006, China;

[3] School of Basic Medical Science, Guangzhou University of Chinese Medicine, Waihuan East Road No. 232, Guangzhou Higher Education Mega Center, Guangzhou 510006, China;

[4] The Research Center of Basic Integrative Medicine, Guangzhou University of Chinese Medicine, Waihuan East Road No. 232, Guangzhou Higher Education Mega Center, Guangzhou 510006, China;

* Correspondence: lixican@126.com or lixc@gzucm.edu.cn (X.L.); chen888@gzucm.edu.cn (D.C.); Tel.: +86-203-935-8076 (X.L.)

† These authors contributed equally to this work.

Suppl. 1 **The structures of ABTS·+, PTIO·, and DPPH·**

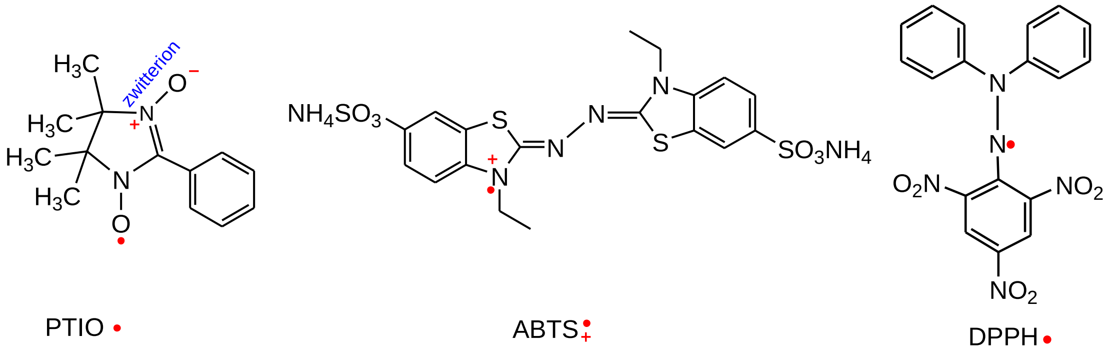

Fig. S1 The structures of ABTS·+, PTIO·, and DPPH·

Suppl. 2 The experimental results of DPPH· scavenging assay

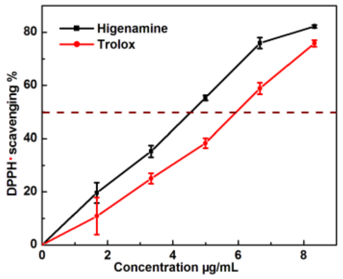

Fig. S2 The dose response curves of DPPH· scavenging assay; Each value is expressed as mean ± SD, n = 3

Table S1 The comparison of IC_{50} values of higenamine and Trolox in free radical assays.

	**higenamine**	**Trolox**
DPPH• scavenging assay μg/mL	4.6±0.5	5.3±0.1
DPPH• scavenging assay μM	17.1±0.2	23.3±0.3

The data underlined were cited in Fig. 2 in the main text.

Suppl. 3 The original data of UPLC-ESI-Q-TOF-MS/MS analysis

Higenamine standard

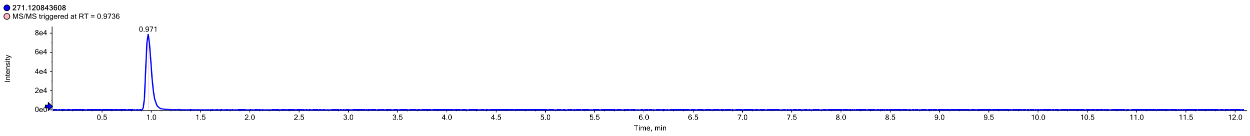

Fig. S3 UPLC chromatography diagram of higenamine standard

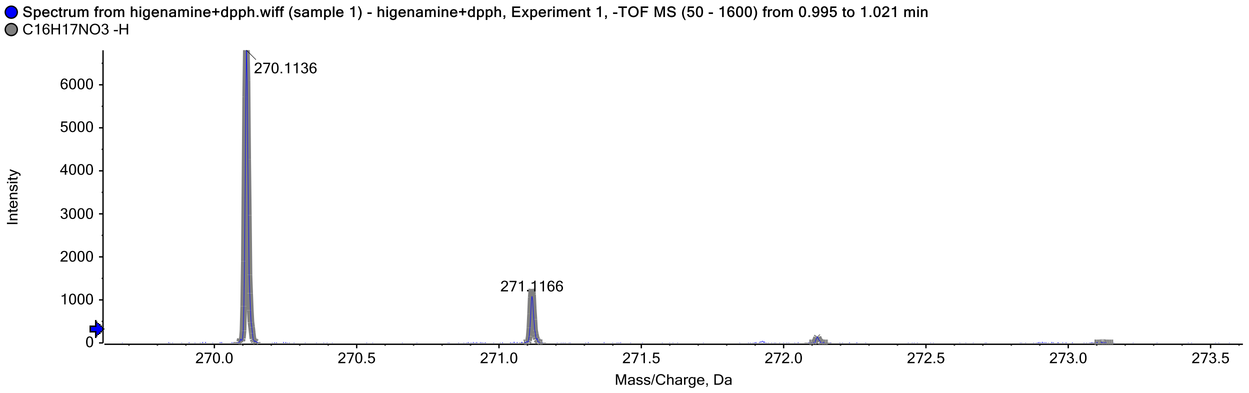

Fig. S4 MS spectra of higenamine standard

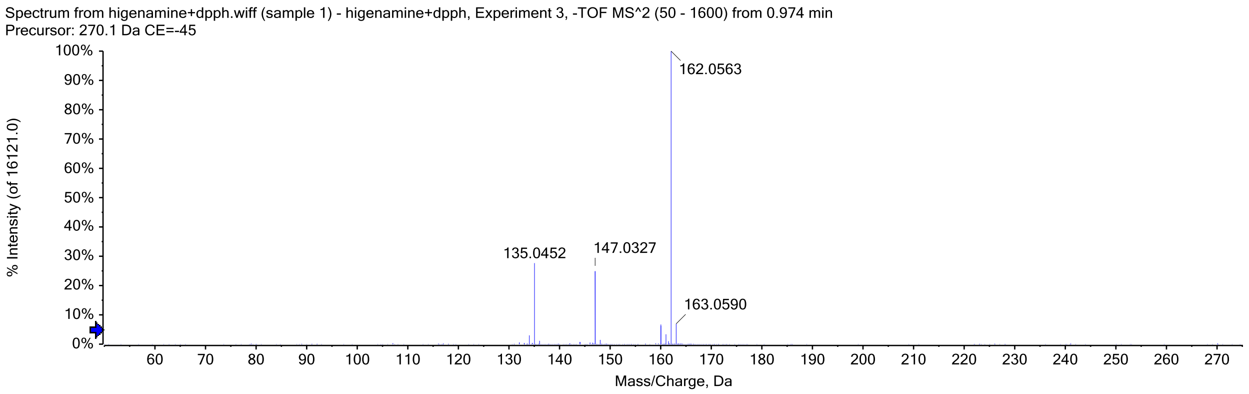

Fig. S5 MS/MS spectra of higenamine standard

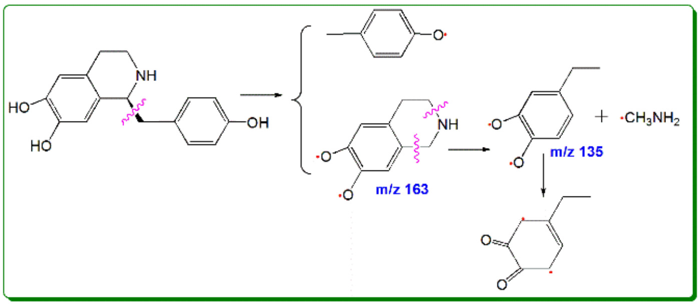

Fig. S6 The possible fragments of higenamine standard

DPPH• standard

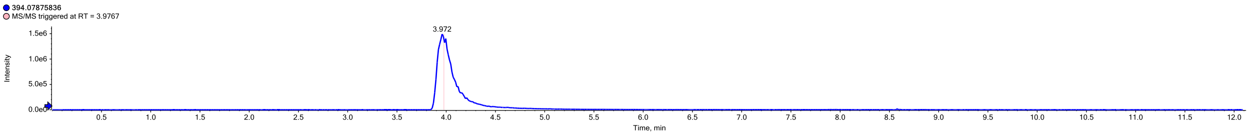

Fig. S7 UPLC chromatography diagram of DPPH• standard

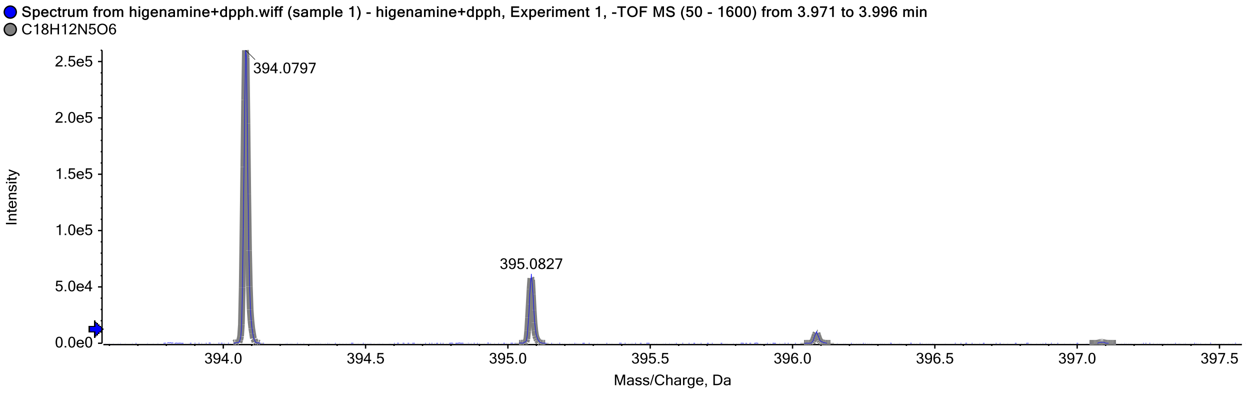

Fig. S8 MS spectra of DPPH• standard

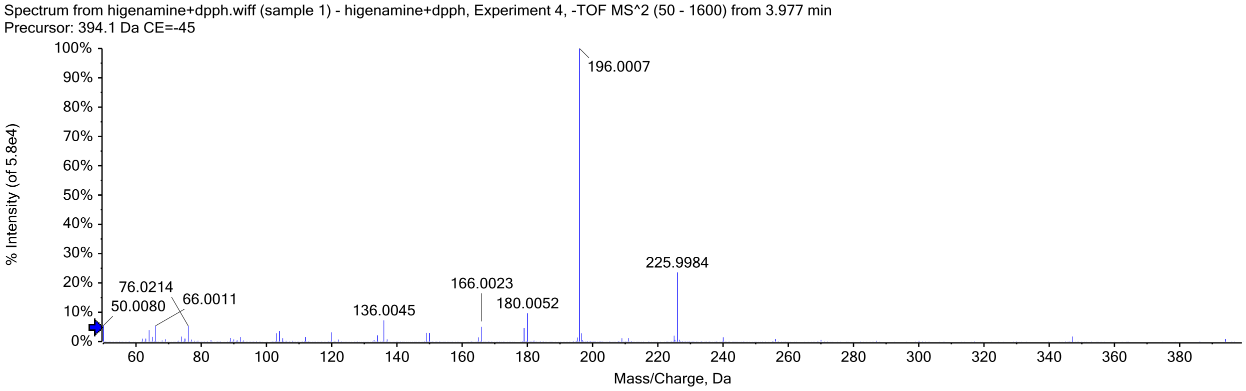

Fig. S9 MS/MS spectra of DPPH• standard

Higenamine-Higenamine ($C_{32}H_{34}N_2O_6$-H)

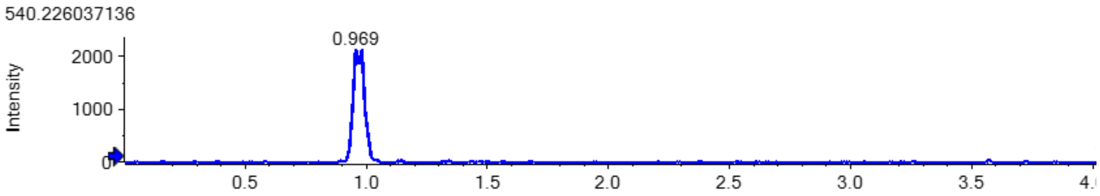

Fig. S10 UPLC chromatography diagram of higenamine–higenamine dimer

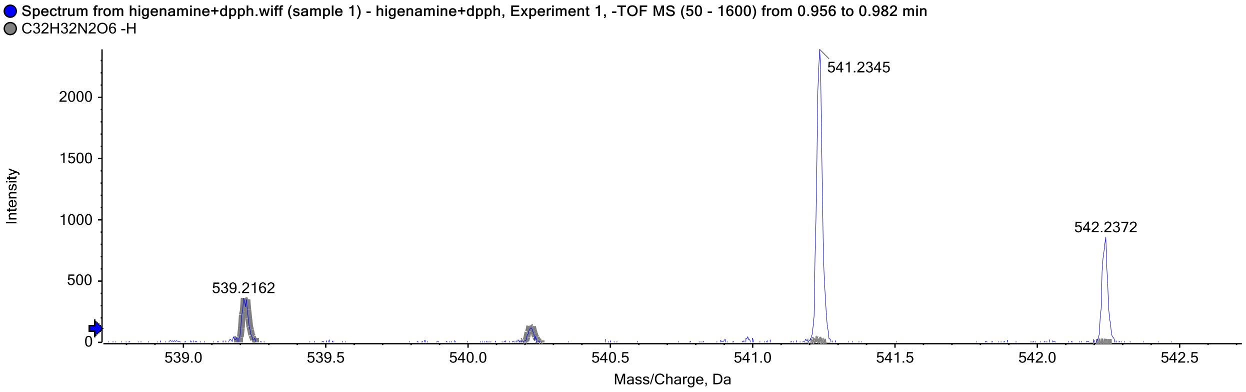

Fig. S11 MS spectra of higenamine–higenamine dimer

Higenamine–DPPH ($C_{34}H_{28}N_6O_9$-H)

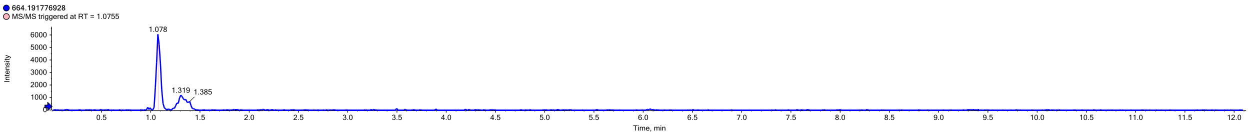

Fig. S12 UPLC chromatography diagram of higenamine–DPPH adduct

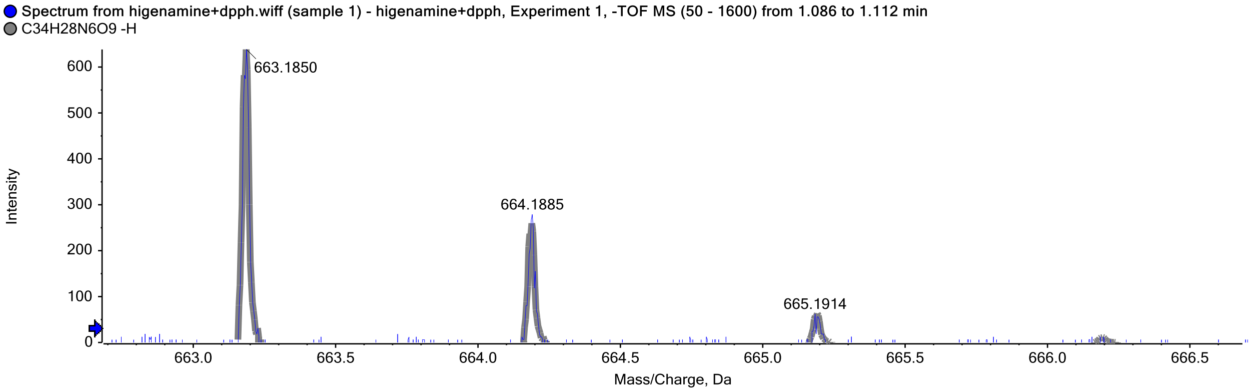

Fig. S13 MS spectra of higenamine–DPPH adduct

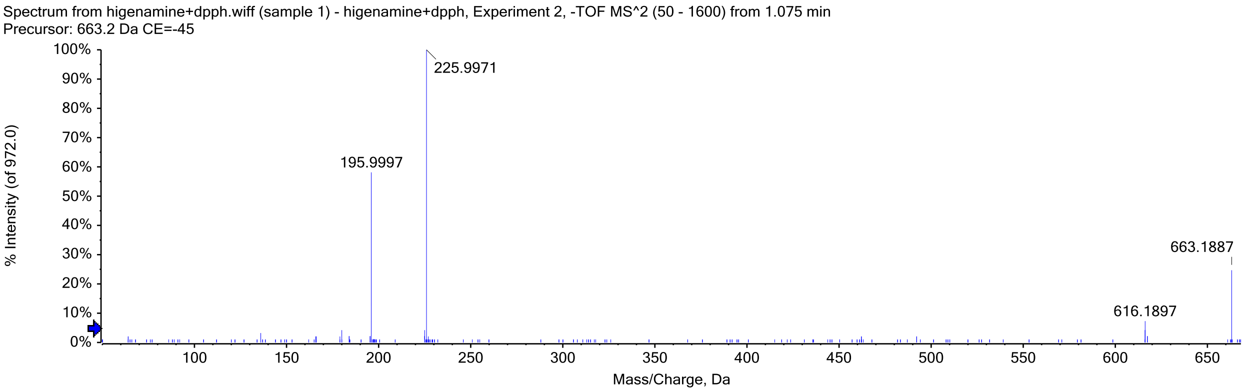

Fig. S14 MS/MS spectra of higenamine–DPPH adduct

Suppl. 4 **The experimental results of PTIO• assay**

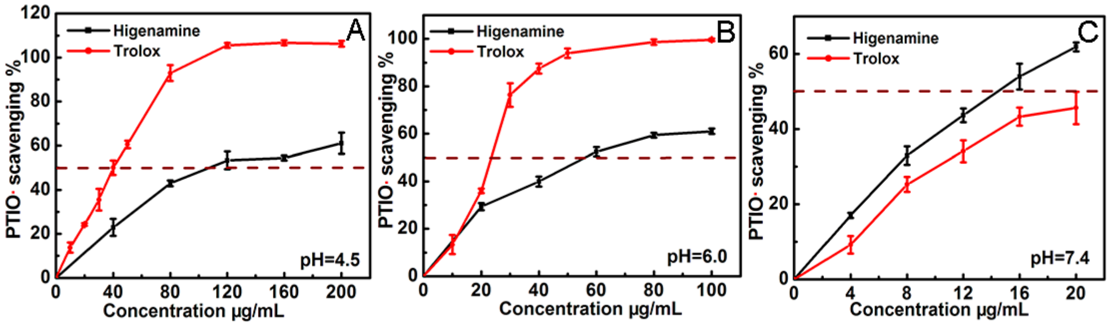

Fig. S15 A The dose response curves of PTIO• (pH 4.5) assays; B The dose response curves of PTIO• (pH 6.0) assays; C The dose response curves of PTIO• (pH 7.4) assays; Each value is expressed as mean ± SD, n = 3

Table S2 The comparison of IC_{50} values of higenamine and Trolox in PTIO•-scavenging.

	**higenamine**	**Trolox**
PTIO•-scavenging (pH 4.5) μg/mL	104.8 ± 6.5	41.1 ± 2.3
PTIO•-scavenging (pH 4.5) μM	386.5 ± 24.1	164.2 ± 9.1
PTIO•-scavenging (pH 6.0) μg/mL	53.4 ± 2.6	25.1 ± 0.7
PTIO•-scavenging (pH 6.0) μM	196.6 ± 9.4	100.1 ± 2.8
PTIO•-scavenging (pH 7.4) μg/mL	14.9 ± 0.4	20.3 ± 1.4
PTIO•-scavenging (pH 7.4) μM	55.0 ± 1.7	81.0 ± 5.6

The data underlined were cited in Fig. 4 or Table 1 in the main text.

Suppl. 5 The experimental results of Fe^{3+}-reducing and Cu^{2+}-reducing assays

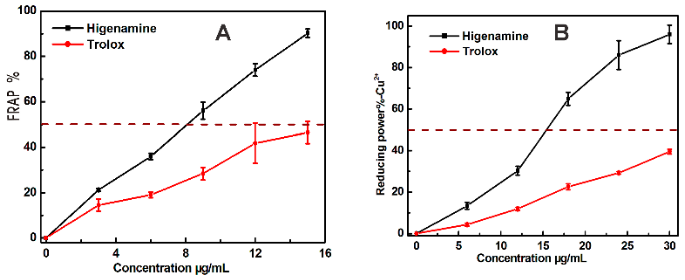

Fig. S16 A The dose response curves of Fe^{3+}-reducing assay (FPRP); B The dose response curves of Cu^{2+}-reducing assays; Each value is expressed as mean ± SD, n = 3

Table S3 The comparison of IC_{50} values of higenamine and Trolox in Metal-reducing assay.

	**higenamine**	**Trolox**
Fe^{3+}-reducing assay (FRAP) μg/mL	8.1±0.1	15.8±2.0
Fe^{3+}-reducing assay (FRAP) μM	30.0±0.3	63.1±8.0
Cu^{2+}-reducing assay μg/mL	15.5±0.2	35.5±1.1
Cu^{2+}-reducing assay μM	57.0±1.0	154.8±2.0

The data underlined were cited in Table 1 in the main text.

Suppl. 6 The analysis certificate of higenamine

四川省维克奇生物科技有限公司
Sichuan Weikeqi Biological Technology Co., Ltd.

质检报告

Quality Test Report

<table>
<tr><td>产品名称</td><td colspan="2">去甲乌药碱</td><td colspan="3" rowspan="10">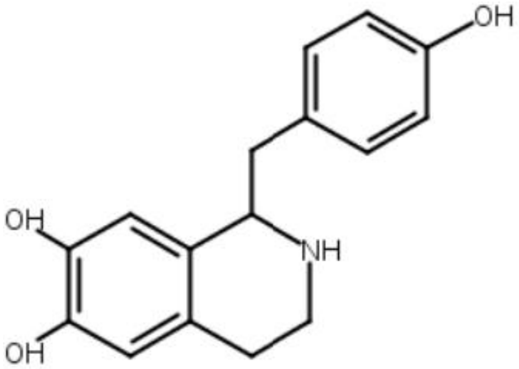
</td></tr>
<tr><td>Product Name</td><td colspan="2">Higenamine</td></tr>
<tr><td>数量</td><td colspan="2">26.98g</td></tr>
<tr><td>Amount</td><td colspan="2">26.98g</td></tr>
<tr><td>批号</td><td colspan="2">wkq16060301</td></tr>
<tr><td>Batch Number</td><td colspan="2">wkq16060301</td></tr>
<tr><td>报告日期</td><td colspan="2">2016.06.03</td></tr>
<tr><td>Report Date</td><td colspan="2">2016.06.03</td></tr>
<tr><td>生产日期</td><td colspan="2">2016.05.24</td></tr>
<tr><td>Manufacture Date</td><td colspan="2">2016.05.24</td></tr>
<tr><td>分子式</td><td>$C_{16}H_{17}NO_3$</td><td>分子量</td><td>271.314</td><td>CAS 号</td><td>5843-65-2</td></tr>
<tr><td colspan="2">项目</td><td colspan="2">规定</td><td colspan="2">结果</td></tr>
<tr><td colspan="2">Characters</td><td colspan="2">Provisions</td><td colspan="2">Results</td></tr>
<tr><td colspan="2">性状</td><td colspan="2">白色粉末</td><td colspan="2">符合</td></tr>
<tr><td colspan="2">Characteristics</td><td colspan="2">White powder</td><td colspan="2">Complies</td></tr>
<tr><td colspan="2">纯度 Purity(HPLC)</td><td colspan="2">≥98%</td><td colspan="2">98.36%</td></tr>
<tr><td colspan="2">Analyst:</td><td colspan="2">Checker:</td><td colspan="2">Q.C.director</td></tr>
<tr><td colspan="2">检验员:</td><td colspan="2">复核员:</td><td colspan="2">负责人</td></tr>
</table>

中文网站：www.weikeqi-biotech.com Official Web: http://www.weikeqi-biological.com
邮箱：scwkqzj@scdzp.com International Mail: scwkqlql@scdzp.com
Tel：0086-028-81700200 4008005713 Fax：0086-028-81705658
Office Address: Room 3,20th floor,Jinsha Lansheng, Middle Qingjiang Road No. 63,Qingyang District, Chengdu, Sichuan.
Lab Address：8th floor, Chengdu Institute of Biology, Chinese Academy of Sciences

去甲乌药碱液相图谱

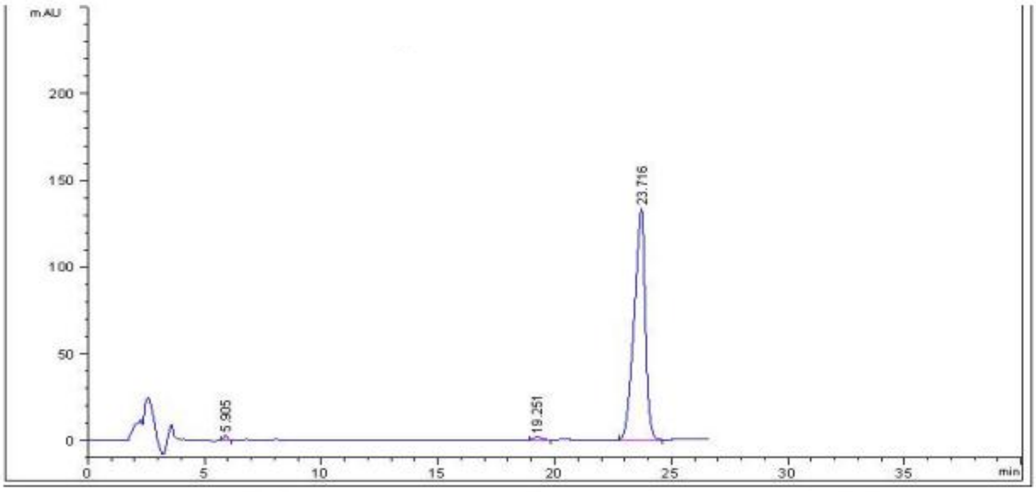

峰 #	保留时间 [min]	类型	峰宽 [min]	峰面积 mAU *s	峰高 [mAU]	峰面积 %
1	5.905	BB	0.1586	30.17862	2.88027	0.6748
2	19.251	BB	0.3695	42.97988	1.79607	0.9610
3	23.716	BB	0.4928	4399.30713	133.28027	98.3642
总量 :				4472.46563	137.95661	
